# Obesity-Induced Hyperglycemia and Heart Failure Preserved Ejection Fraction: Uncharted Territories to Remission

**DOI:** 10.7759/cureus.49178

**Published:** 2023-11-21

**Authors:** Khalid Sawalha, Osama Asad, Shourya Tadisina, Luay Alalawi, Maria Mahmood, Deya Alkhatib, Thomas Alexander

**Affiliations:** 1 Cardiometabolic Medicine, University of Missouri Kansas City School of Medicine, Kansas City, USA; 2 Internal Medicine, Jordan University of Science and Technology, Irbid, JOR; 3 Endocrinology, Diabetes and Metabolism, University of Missouri Kansas City School of Medicine, Kansas City, USA; 4 Cardiology, Corpus Christi Medical Center Bay Area, Corpus Christi, USA; 5 Marvin Baker Middle School, N/A, Corpus Christi, USA; 6 Cardiology, Yale School of Medicine, New Haven, USA; 7 Cardiology, Corpus Christi Medical Center, Corpus Christi, USA

**Keywords:** cardiometabolic diseases, cardiovascular disease prevention, hyperglycemia, preventative cardiology, reversible heart failure, obesity induced heart failure preserved ejection fraction, obesity induced hyperglycemia

## Abstract

Until the end of World War II, food security was a global challenge. Consequently, in 1948, type 2 diabetes was relatively uncommon, with the majority of cases being type 1 diabetes requiring insulin therapy. Since then, food has become increasingly palatable and readily available, leading to a rise in obesity across all age groups. Understanding the impact of obesity on our health has become crucial for optimizing healthcare. In this context, we draw attention to two significant, yet relatively uncharted pathogenic effects associated with obesity: Hyperglycemia and Heart Failure with Preserved Ejection Fraction (HFpEF). Thorough pathophysiologic, hemodynamic, and echocardiographic characterization have revealed the existence of a distinct phenotype known as “obese HFpEF” within the broader HFpEF population, and “obesity-induced hyperglycemia” within the diabetes population. In these phenotypes, patients often present with higher Body Mass Index and experience clinical symptoms decades earlier. Recent insights have enhanced our understanding of the mechanisms underlying obesity-mediated heart failure preserved ejection fraction and hyperglycemia. Early detection offers the potential for reversibility of many pathologies associated with obesity through adequate weight reduction. The objective of this review is to provide a deeper insight into these uncharted territories and explore the potential for improved outcomes by reframing these two narratives toward achieving remission. Such a shift has the potential to positively impact individual engagement with healthier lifestyles.

## Introduction and background

Over the past few decades, the prevalence of obesity has reached alarming levels and is considered a global health epidemic, particularly in the United States [1]. Obesity is associated with numerous cardiovascular risk factors, including hypertension, type 2 diabetes, and dyslipidemia [2]. However, recent insights have enhanced our understanding of the mechanisms underlying the development of obesity-related complications, suggesting significant interindividual heterogeneity, particularly including obesity-induced hyperglycemia (OH) and obesity-induced heart failure (HF) with preserved ejection fraction [3,4].

Body mass index (BMI) is a widely used tool for screening for overweight and obesity in adults and children [5]. It is defined as weight in kilograms divided by height in meters squared and is related to the amount of fat in the body [5]. BMI classifies obesity into three classes: class 1 where the BMI is 30 to less than 35 kg/m², class 2 with a BMI of 35 to less than 40 kg/m², and class 3 with a BMI of 40 or higher kg/m², sometimes categorized as “severe” or “morbid” obesity [5]. Although it is widely used as a screening tool, it is worth mentioning that it does not reflect total body composition or body fatness [5].

Having those clear definitions in mind, it is projected that by 2030 in the US, the prevalence of class 2 obesity will likely reach 25% and the diabetes prevalence to 13.9% [6,7]. This affects individuals of all ages, genders, and socioeconomic statuses. This prevalence not only poses a significant burden on healthcare systems but also increases the risk of various cardiometabolic diseases and certain types of cancer [8]. Therefore, broadening our understanding and changing our approach regarding obesity-related complications could offer significant health benefits. Our future is determined by how we approach things now.

## Review

Obesity-induced hyperglycemia

OH is an emerging clinical phenotype characterized by elevated blood glucose levels resulting from excess body fat accumulation [3]. It is a consequence of the intricate relationship between adipose tissue, hormones, and insulin resistance [9]. As we gain excess weight, our body's ability to regulate blood glucose becomes impaired, leading to higher blood glucose levels [9]. It is usually associated with BMI >35 kg/m² and diabetes. Understanding the mechanisms behind OH is vital for developing effective strategies for prevention and management.

The pathophysiology of OH is still not completely understood, but it is thought to result from derangements in insulin signaling pathways, along with other etiological factors [10]. Obesity causes an increase in insulin resistance and beta cell dysfunction through the induction of inflammation, endocrinopathies, and increased circulating free fatty acids (FFA) [10,11]. It also increases pro-inflammatory macrophages, which are recruited in response to adipocyte apoptosis, leading to an increased release of inflammatory cytokines like tumor necrosis factor-α (TNF-α), interleukin-6, and interleukin-1β, causing a state of chronic inflammation. Peroxisome proliferator-activated receptor gamma (PPAR-γ) also plays an important role in regulating insulin sensitivity, adipogenesis, glucose, and lipid homeostasis [10,11]. TNF-α can decrease PPAR-γ, insulin receptors, insulin receptor substrate, glucose transporters, and adipose tissue functionality. It causes an increase in lipolysis, resulting in increased delivery f circulating FFAs to the liver, muscle, and pancreas [10,11]. This increase in FFA mediates mitochondrial dysfunction and increases reactive oxygen species, decreasing the phosphorylation of insulin receptor substrates and impairing insulin receptor activity [11]. An increase in IL-6 production leads to increased hepatic C-reactive protein (CRP) production. Obesity can also decrease adiponectin, an anti-inflammatory cytokine produced by adipocytes that improves insulin sensitivity [11].

Leptin is a hormone secreted by adipocytes and works on the hypothalamus to suppress hunger [10-12]. Chronic leptin stimulation of the arcuate nucleus of the hypothalamus can, in some cases, promote the protein tyrosine phosphatase 1B (PTB1B), which inhibits insulin activity [11]. Leptin lowers blood glucose levels and has anti-lipogenic effects. Obesity can cause an increase in both leptin and insulin levels, contributing to insulin resistance [11]. Acylation-stimulating protein (ASP) is secreted by adipocytes, acting as a lipogenic factor that promotes triglyceride synthesis and storage in adipocytes. A relative decrease in ASP can occur in obesity, leading to a defect in glucose and lipid metabolism [11]. 11 beta-hydroxysteroid dehydrogenase type-1 (11βOHSD-1) is an enzyme produced by adipose tissue and the liver, which converts inactive cortisone to active cortisol and is increased with obesity [11]. It amplifies the glucocorticoid effects even when the glucocorticoid levels are normal. This can cause increased lipolysis and the release of FFA, an increase in gluconeogenesis, and a decrease in peripheral glucose uptake as well [11,12].

Lipotoxicity from increased FFA can also cause hyperglycemia and eventually diabetes [10]. Impaired uptake of energy in adipose tissue can lead to an increase in circulating FFAs, resulting in ectopic and pathogenic fat deposition in the visceral, pericardial, perivascular regions, liver, muscles, and pancreas [11,12]. In the liver and muscles, an increase in intracellular binding of FFAs and sphingolipids forms toxic ceramides and their metabolites, leading to mitochondrial dysfunction, endoplasmic reticulum stress, impaired insulin receptor function, and impaired expression of glucose transporters. In the pancreas, increased delivery of FFAs causes Lipotoxicity, leading to beta cell apoptosis and decreased insulin secretion [10,11].

By now, we have established the possible pathophysiologies associated with OH. We know that type 1 diabetes is a genuine endocrine disorder that develops as a result of autoimmune beta cell dysfunction, leading to insulin deficiency [12]. On the other hand, type 2 diabetes results from insulin resistance caused by genetic and environmental factors [12]. OH shares similar etiologies with type 2 diabetes, except that it occurs before the development of type 2 diabetes and involves experiencing a state of chronic hyperinsulinemia for decades [3,12]. Further differences and comparisons between type 1, type 2, and OH can be seen in Table 1.

**Table 1:** Overview of type 1, type 2, and obesity-induced hyperglycemia. Author's own work [3].

Criteria/Type	Type 1 diabetes	Type 2 diabetes	Obesity-Induced Hyperglycemia
Definition	Insulin deficiency	Insulin resistance	BMI >35 kg/m^2^ and hyperglycemia
Etiology	Autoimmune leading to Insulin deficiency	Genetics and environmental leading to Insulin resistance	Diet and sedentary lifestyles leading to excess endogenous insulin secretion for decades
Prevalence	< 5–10%	> 95% of all diabetics	30–35% of patients with type 2 diabetes
Symptoms onset	At diagnosis	Delayed by years	Delayed by decades
Significant weight loss manifested by >20%	Will continue to need insulin therapy but probably less amounts	Significant reduction in medication requirement and number	Often may be able to discontinue all medication
Management	Insulin	Diet, lifestyle, and medications	Lifestyle and diet changes
Prognosis	Lifetime management	Variable	High remission with possible cure

Lifestyle modifications and significant weight loss of more than 20% of body weight have demonstrated a significant impact on treating and reversing OH [13]. The same degree of weight loss can improve insulin sensitivity and reduce medication requirements in patients with type 2 diabetes, although it does not guarantee complete resolution [13]. With appropriate lifestyle modifications, OH can be reversible and potentially curable, thus enhancing life expectancy and reducing healthcare expenses [14].

Obesity-induced HFpEF

Over the past two decades, we have witnessed a shift in the paradigm of HFpEF, challenging not only our understanding of its pathophysiology but also our approaches to diagnosis and treatment. HFpEF should no longer be viewed solely as left ventricular diastolic dysfunction [15]. Instead, it should be considered a condition characterized by small and thickened left ventricles, exhibiting abnormal diastolic filling patterns as its primary pathophysiological abnormality. This heterogeneous clinical entity encompasses numerous mechanisms and is associated with multiorgan dysfunction, with hypertension and obesity playing significant roles [15].

Obesity-induced cardiomyopathy (OC) is caused by an increase in total blood volume, cardiac output, left ventricular dilatation, and left ventricular wall stress [15,16]. It can also induce diastolic dysfunction through compensatory left ventricular hypertrophy [15,16]. This condition is primarily attributed to the hyperdynamic state and chronic inflammation resulting from OH and Lipotoxicity [16,17]. In the absence of other comorbidities, young adults with morbid obesity which is defined as a body mass index or BMI of > 40 kg/m² can develop OC. This condition may be reversible, as significant weight loss achieved through gastric bypass surgery can lead to improvements in cardiac dimensions [4,18].

Systolic dysfunction is sometimes observed in association with obesity, but it is important to note that other confounding factors such as obstructive sleep apnea, atrial fibrillation, and hypertension cannot be ruled out [17,19]. The structural changes in cardiomyopathy due to obesity are categorized as stage B HF according to the ACC/AHA classification. Typically, these patients do not present in a decompensated state [20].

The duration of obesity significantly determines the cardiac burden. The likelihood of developing obesity cardiomyopathy increases from 20% at 15 years of obesity duration to 95% at 25 years [21]. However, it is often challenging for patients to accurately recall their weight history over the years, making it difficult to predict the exact burden. “Obesity-years” is a term that has been suggested to estimate the cumulative burden of obesity over a person's lifetime [4].

In a study comparing cohorts of individuals with typical HFpEF, obese HFpEF, and obesity cardiomyopathy, data was charted on a graph with age on the X-axis and BMI on the Y-axis [22,23]. Interestingly, the line representing Age + BMI = 100 intersects with all these cohorts [22,23]. This suggests that it might serve as an arbitrary method for assessing the likelihood of developing HF [22,23].

HF of obesity (HFO) is a relatively other new term used to describe HF in obese individuals, independent of other comorbidities [4,24]. This includes obese HFpEF patients in stage C HF (decompensated) and patients with obesity cardiomyopathy in stage B HF (structural heart disease or diastolic dysfunction) [24]. HFO is likely to develop by age 30 with a BMI >70 kg/m^2^ and by age 50 in asymptomatic obese individuals with a BMI of 50 kg/m^2^ [4]. Clinical diagnosis of HFO is challenging since HF symptoms can be attributed to deconditioning resulting from obesity, the elevation of brain natriuretic peptide (BNP) is blunted, and weight limits cardiac catheterization and evaluation by echocardiography [24]. In HFO, both stage B and stage C fall along a continuum of compensatory cardiac adaptations to the excess workload. The likelihood of stage B increases as age + BMI approaches 100 [4]. Stage C HFO requires signs of decompensation, which include volume overload, pulmonary congestion, or the need for diuretics [4,24].

Cardiac resilience is the ability to maintain adequate cardiac output despite comorbidities (such as hypertension and valve disease) and acute stressors (such as surgery or infection) [24,25]. This resilience tends to decline with aging [25]. Lifestyle modifications can improve resilience and reverse biological aging [26,27]. In HFO, the adaptive cardiac changes are mediated by hemodynamic load and inflammation. Patients who achieve a >20% weight reduction by the age of 60-70 years can experience complete reversal of HF [26]. Therefore, educating patients about “Obesity-years” will benefit from cardiac evaluation.

Future studies are in critical need to support a paradigm shift in our approach to obesity-related complications, emphasizing the potential for remission through targeted interventions and lifestyle modifications, ultimately leading to improved health outcomes for affected individuals.

## Conclusions

In summary, OH emerges as a consequence of the intricate interplay between adipose tissue, hormones, and insulin resistance, resulting in elevated blood glucose levels. Understanding its underlying mechanisms is pivotal for effective prevention and management. Importantly, OH presents a unique opportunity for reversal through substantial weight loss and lifestyle modifications, potentially offering a path to remission that can alleviate the burden on healthcare systems and improve the quality of life for affected individuals. On the other hand, recognizing the heterogeneity of HFpEF and its association with obesity underscores the importance of tailored diagnostic and therapeutic approaches. Moreover, early intervention through weight reduction interventions may offer a promising avenue for reversing cardiac changes, thus potentially mitigating the progression of HFpEF.
